# Fabrication of Potassium- and Rubidium-Doped Formamidinium Lead Bromide Nanocrystals for Surface Defect Passivation and Improved Photoluminescence Stability

**DOI:** 10.1021/acsaelm.3c01542

**Published:** 2024-01-04

**Authors:** Madeeha Tabassum, Qasim Zia, Huanqing Ye, William George Neal, Sameen Aslam, Jinshuai Zhang, Lei Su

**Affiliations:** † Key Laboratory of Advanced Materials and Nanotechnology, School of Engineering and Materials Science, Queen Mary, University of London, London E14NS, U.K.; ‡ NanoVision Centre for Structural and Chemical Analysis, School of Engineering and Materials Science, Queen Mary, University of London, London E14NS, U.K.; § The Photon Science Institute, Department of Electrical and Electronic Engineering, University of Manchester, Manchester M13 9PY, U.K.; ∥ Centre for Condensed Matter and Materials Physics, School of Physical and Chemical Sciences, Queen Mary, University of London, London E14NS, U.K.; ⊥ Garments Technology Department, Punjab Tianjin University of Technology, Lahore 53720, Pakistan; # Key Laboratory of Nanophononics and Semiconductor Optics, Materials Science and Engineering, Peking University, Yiheyuan Road, Beijing 100871, China

**Keywords:** halide perovskite nanocrystals, surface defects, colloidal stability, alkali metal ions, doping

## Abstract

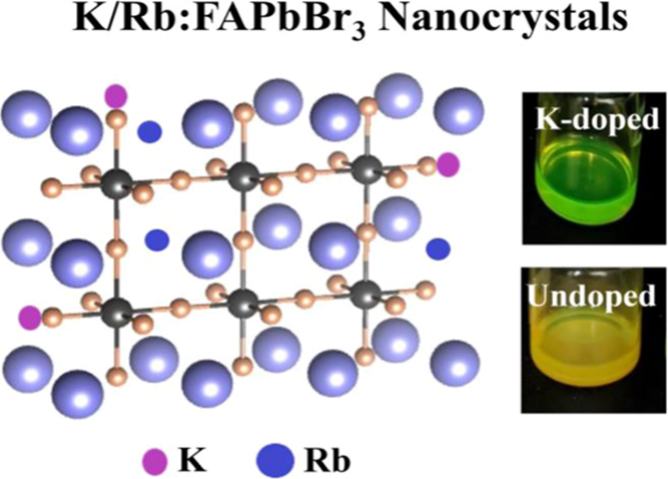

The past decade has seen a rapid development in metal halide perovskite nanocrystals (NCs), which has been witnessed by their potential applications in nanotechnology. The inimitable chemical nature behind their unique photoluminescence characteristics has attracted a growing body of researchers. However, the low intrinsic stability and surface defects of perovskite NCs have hampered their widespread applications. Therefore, numerous techniques such as doping and encapsulation (polymer matrices, silica coating, salt matrix, etc.) have been examined for the surface modification of perovskite NCs and to increase their efficiency and stability. In this study, we demonstrated the self-passivation method for surface defects by introducing potassium (K) or rubidium (Rb) during the colloidal fabrication of NCs, resulting in the much-improved crystallinity, photoluminescence, and improved radiative efficiency. In addition, K-doped NCs showed a long-term colloidal stability of more than 1 month, which indicates the strong bonding between the NCs and the smaller-sized potassium cations (K^+^). We observed the enhancement of the radiative lifetime that can also be explained by the prevention of “Frenkel defects” when K^+^ stays at the interstitial site of the nanocrystal structure. Furthermore, our current findings signify the importance of surface modification techniques using alkali metal ions to reduce the surface traps of perovskite nanocrystals (PeNCs). Comparable developments could be applied to polycrystalline perovskite thin films to reduce the interface trap densities. The findings of this study have several important implications for future light-emitting applications.

## 1 Introduction

In the past few years, metal halide perovskite nanocrystals (MHP NCs) have received considerable attention owing to their high color purity, enhanced photoluminescence (PL) properties, and readily tunable band gaps from the visible to the near-infrared spectral region through halide exchange or size control.^1^ Perovskite NCs have emerged as materials of choice because of their predominantly ionic lattice and low-temperature synthesis, and hence they are readily precipitated via low-cost solution processes.^2^ So far, formamidinium lead bromide (FAPbBr_3_) NCs have arrested outstanding photophysical properties such as stable and ultrapure green emission with tunable PL (525–535 nm), small full width at half-maxima (fwhm < 25 nm), and excellent stability in the air at high temperatures. In contrast, the most popular methylammonium lead bromide (MAPbBr_3_) and cesium lead bromide (CsPbBr_3_) have shown difficulties in achieving the desirable “525–535” PL defined by the “International Telecommunication Union (ITU) Recommendation BT 2020 (Rec. 2020) standard” for next-generation displays. Therefore, FAPbBr_3_ NCs can ideally realize the critical window of the “Rec. 2020 standard” for ultrapure green emitters.^3^

Metal halide perovskites (MHPs) crystallize in the cubic ABX_3_-type perovskite structure, where A is a cation (for example, methylammonium (CH_3_NH_3_^+^), formamidinium (CH(NH_2_)_2_^+^/FA^+^), or cesium (Cs^+^)), B is a divalent metal cation (mostly lead (Pb^2+^)), and X is one or more halide anions (Cl^–^, Br^–^, I^–^).^4^ In the form of colloidal NCs, these materials are terminated by long-chain alkyl ligands that contain ionic substituents such as an amine cation (R-NH_3_^+^) and a carboxylate anion (R-COO^–^) to achieve a stable colloidal solution in nonpolar solvents. However, these ligands attach to the boundary of perovskite NCs by weak electrostatic forces and only provide high coverage when present in excess in solution. In addition, the proton transfer between cationic amines and anionic carboxylates generates deprotonated NH_2_ and protonated COOH functional groups, leading to the rapid removal of ligands from the outer surface of NCs during the purification and storage process. The main consequences of such a weak binding interaction between NCs and ligands are that it decreases luminance efficiency and colloidal solution stability by the incomplete surface passivation of NC sites or defects.^5,6^

A high photoluminescence quantum yield (PLQY) and long-term structural stability of MHP NCs are fundamental requirements for commercial applications in television displays and related devices. However, surface defects of MHP NCs have accentuated the problem of charge-trapping and substantially increased the number of trap-assisted carrier recombination pathways, reducing the performance of NCs for desired applications. To date, researchers have been able to explore various techniques to overcome the problem of long-term NC stability including postsynthetic surface treatment,^5^ doping into NCs with metal and rare earth ions,^7,8^ and reducing defects using surface-capping methods.^9^ In particular, many studies have established that the addition of alkali metal ions to perovskite NCs can significantly tailor the properties of this fascinating material. K addition was reported to increase the structural rigidity of mixed-halide CsPbBr_1.5_I_1.5_ NCs, which elevates thermal activation energy, hence enabling the NCs to retain a high photoluminescence emission intensity at high temperatures (>353 K).^10^ Another study reported an outstanding external quantum efficiency (EQE) of 21.8% and a lifetime *T*_50_ of 69 min by inserting K as a passivating agent between the emissive layer (CsPbBrI_3_:Sr) and the hole transport layer.^11^ A PL inhibition phenomenon was unveiled by a group of researchers, where PL was first increased by the addition of K into the MAPbBr_3_ NCs and then inhibited with a further amount of K^+^.^12^ Likewise, Rb doping into the perovskite NCs improves the crystallinity and reduces the nonradiative traps, in that way improving the thermal behavior of doped NCs. Manganese (Mn)-doped CsPbCl_3_ NCs with the addition of Rb^+^ were studied by measuring optoelectronic properties. The experimental findings showed that the Mn^2+^ cation emission for Mn-doped CsPbCl_3_ NCs enhanced after the doping of Rb^+^.^13^ Furthermore, Rb was explored to study the dynamics of carriers in CsPbBr_3_ perovskite single crystals for efficient X-ray detection. This work has revealed that a small amount (0.037%) of Rb incorporation can increase the atomic interaction and orbital coupling between Br and Pb atoms, thereby leading to more efficient carrier transport and X-ray detection.^14^ However, few researchers have been able to draw any systematic research on the treatment of organic–inorganic perovskite materials with alkali metal ions. Therefore, perovskite research studies would have been more useful if they focused on studying the effect of K^+^ and Rb^+^ on perovskite nanocrystals to understand these discrepancies.

The present research sets out to investigate the effects of introducing potassium cations (K^+^) and rubidium cations (Rb^+^) during the ligand-assisted reprecipitation (LARP) process for the fabrication of formamidinium lead bromide (FAPbBr_3_) nanocrystals. The introduced alkali metal cations (K^+^/Rb^+^) act as new passivating agents, which not only occupy the places of dangling organic ligands but also reduce the density of surface defects of PeNCs. This study, therefore, set out to suppress the nonradiative recombination of the FAPbBr_3_ NCs, and consequently, NCs with a high photoluminescence quantum yield (PLQY) are obtained. In addition, K^+^/Rb^+^-doped FAPbBr_3_ NCs showed a long-term colloidal stability of more than 1 month, indicating the strong bond between the NCs and the new metal ligands.

## 2 Experimental Section

### 2.1 Materials

Chemicals including lead bromide (PbBr_2_, ≥98%), formamidinium bromide (FABr, ≥98%), rubidium bromide (RbBr, 99.6%), potassium bromide (KBr, ≥99%), oleic acid (technical grade, 90%), oleylamine (technical grade, 70%), dimethylformamide (DMF, 99.8%), chloroform (≥99%), toluene (99.8%), hexane (≥95%), and acetonitrile (99.8%) were purchased from Sigma-Aldrich. All of the chemicals were directly used as received without further purification.

### 2.2 Synthesis of K^+^/Rb^+^-Doped FAPbBr_3_ NCs

The unpassivated FAPbBr_3_ NCs were fabricated by the ligand-assisted reprecipitation (LARP) method as previously reported by Chen et al. with some modifications.^15^

For the synthesis of K^+^- and Rb^+^-passivated NCs, KBr and RbBr powders were first dissolved in a mixed solution of DMSO/DMF (3:2 v/v ratio) to make a stock solution of 0.07 and 0.05 M, respectively. The resulting KBr and RbBr solutions were added separately into the formamidinium precursor solution using a fixed volume of 30 μL. The remaining steps of K^+^/Rb^+^-doped FAPbBr_3_ NCs are the same as those of the synthesis of undoped FAPbBr_3_ NCs.

### 2.3 Characterizations

The steady-state PL spectra of FAPbBr_3_ NCs were recorded with a fluorescence spectrometer (FLS920, Edinburgh Instruments). The NC dispersions in hexane were illuminated using 475 nm light at room temperature. The ultraviolet–visible (UV–vis) absorbance spectrum of the samples was collected over a range of 400–650 nm using a LAMBDA 35 PerkinElmer spectrometer.

Time-resolved PL spectroscopy was performed utilizing a Horiba Triax 550 spectrometer and detected using a Hamamatsu R5509-72 NIR-PMT detector. A 405 nm vortexed diode laser with a pulse width of 5 ns was used to excite the perovskite NCs. The transmission electron microscopy (TEM) and high-resolution TEM (HR-TEM) images of the NCs were taken on a JEOL-JEM TEM machine working at an accelerating voltage of 200 kV with a camera length of 40 cm. The samples for TEM testing were prepared by dropping 45 μL of solution in hexane onto carbon-coated copper grids and were air-dried well. The Fourier transform infrared (FTIR) spectra were recorded on a Burker model FTIR with an ATR accessory over the range of 4000–400 cm^–1^. The X-ray diffraction (XRD) profile of the prepared NCs was measured using a PANalytical XpertPro diffractometer with an angular range of 5 < 2θ < 70°. The spectra were recorded using monochromatic Cu Kα (λ = 1.541 Å) as a radiation source. The X-ray photoelectron spectroscopy (XPS) spectra of the NCs were recorded on a Thermo Scientific Nexsa XPS System using Al Kα X-rays as the excitation source.

## 3 Results and Discussion

The FAPbBr_3_ nanocrystals (NCs) were prepared by a ligand-assisted reprecipitation (LARP) method. The present study utilizes a precursor solution containing a controlled amount of lead bromide (PbBr_2_) and formamidinium bromide (FABr) in dimethylformamide (DMF), as mentioned in a previously published report.^15^

**Figure 1:**
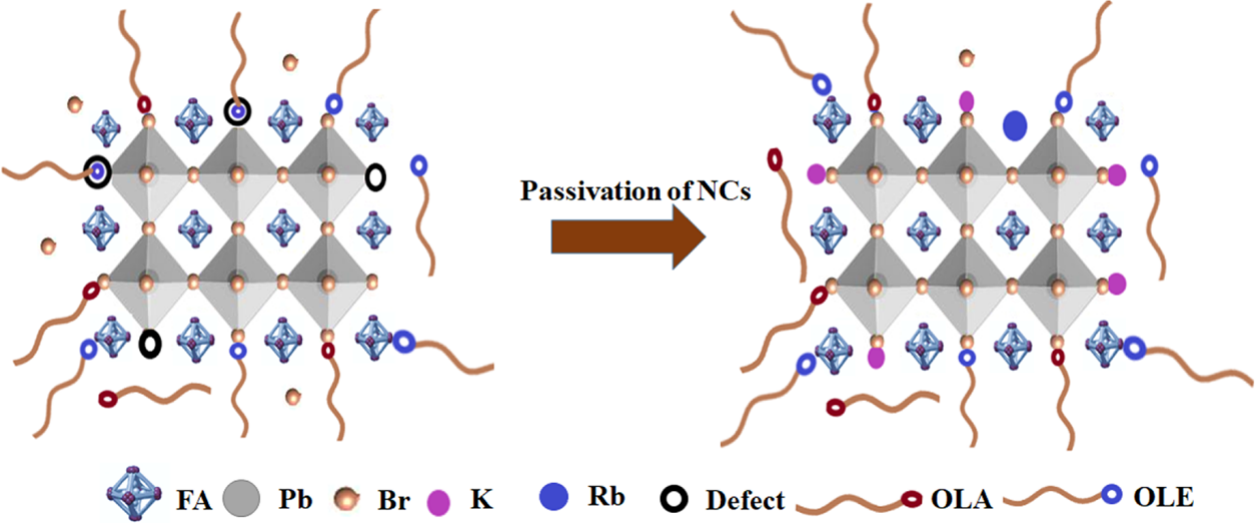
Schematic illustration of potassium and rubidium passivation.

To prepare perovskite nanocrystals (PeNCs) with K and Rb dopants, a controlled amount of KBr and RbBr in DMF was added separately to the formamidinium precursor solution. The doping concentration of K-doped and Rb-doped NCs was controlled by a fixed molar feed ratio of potassium or rubidium to the lead source [K or Rb]/Pb. The added potassium ([K]/[Pb]) and rubidium ([Rb]/[Pb]) concentrations were 5 and 3%, respectively, in the precursor solution. The PeNCs were precipitated with acetonitrile before centrifugation. After purification with hexane several times, the prepared PeNCs were dispersed in hexane for further analysis. To understand the doping behavior of these alkali metal ions, we show a schematic diagram of the treatment technique of K^+^/Rb^+^ in Figure 1.

### 3.1 Structural and Optical Characteristics

**Figure 2:**
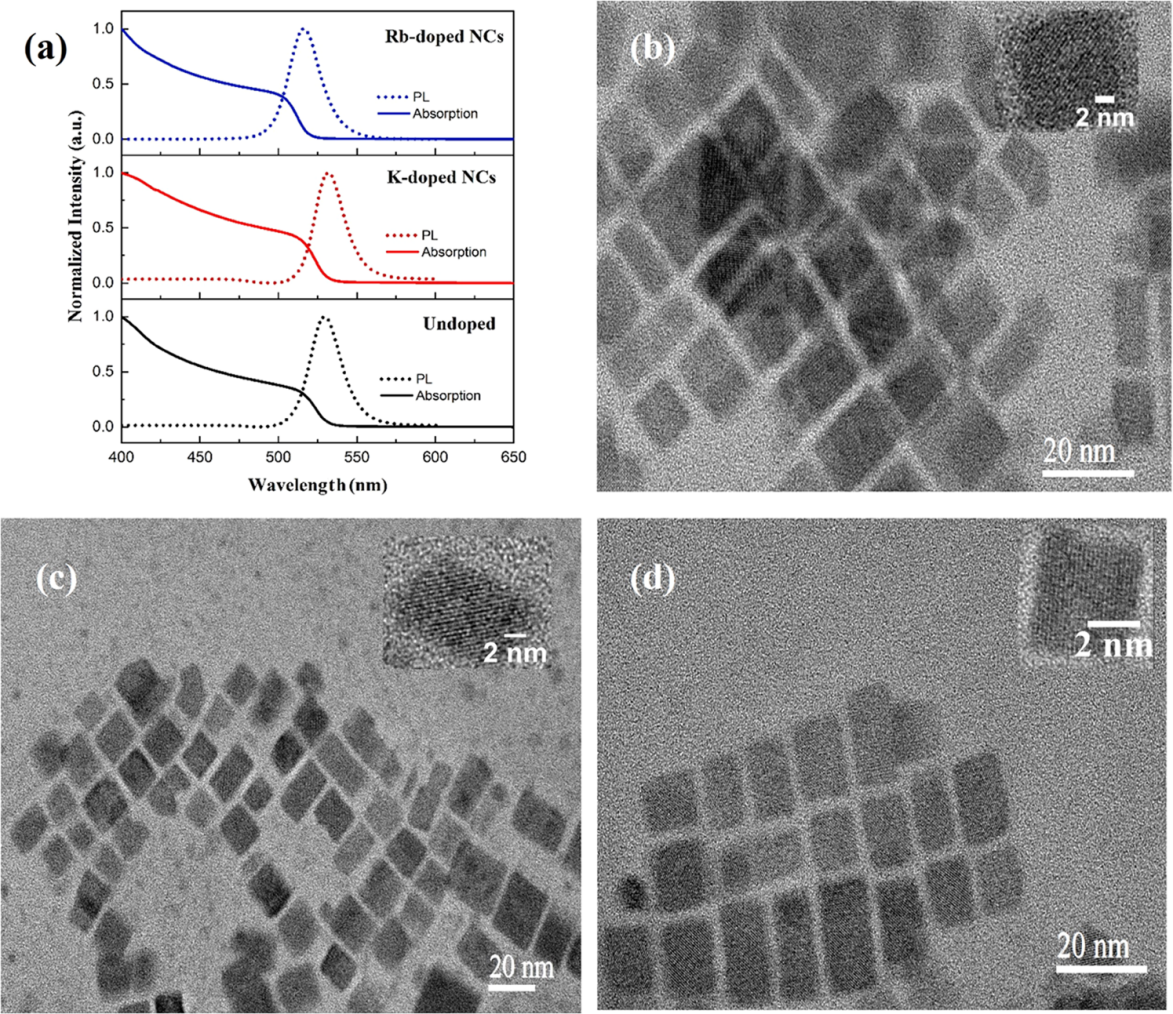
(a) UV–vis absorption and PL spectra. (b–d) TEM images of undoped FAPbBr_3_ NCs, K-doped NCs, and Rb-doped NCs, respectively. The inset shows high-resolution TEM images of the corresponding samples.

The optical properties of the prepared dispersions of FAPbBr_3_ perovskite NCs with and without alkali metal ion [K^+^/Rb^+^] doping were demonstrated by UV–visible absorption and PL characterization. As shown in Figure 2a, the absorbance spectra and the PL spectra show a red shift with K-doped NCs compared to Rb-doped NCs which exhibit a blue shift. Consequently, the band gaps of PeNCs containing different concentrations of alkali metal ions determined by using a Tauc plot of the UV–visible spectrum are 2.23 (undoped), 2.21 (K-doped NCs), and 2.32 (Rb-doped NCs), as can be seen from Figure S1. Table 1 shows a key parameter comparison of the treated and untreated FAPbBr_3_ perovskite NCs.

**Table 1:** Summary of the Characterization of all FAPbBr_3_ Perovskite NC Samples

sample	λ_Abs_ [nm]	λ_PL_ [nm]	energy gap [eV]	fwhm [nm]	NC size [nm]
undoped	532	529	2.23	23	10.72
K-doped NCs	536	532	2.21	22	12.36
Rb-doped NCs	520	516	2.32	23	9.58

A slight decrease in the optical band gap for K-doped nanocrystals may be due to the fabrication of larger NCs caused by the reduced number of organic ligands, as mentioned in the previous study.^16^ For Rb-doped NCs, the broadening of the optical band gap could be the result of an orientational or electrostatic disorder as well as the fabrication of smaller NCs.^17^

The morphology of the K^+^/Rb^+^-doped NCs was described by transmission electron microscopy (TEM). TEM images showed the cubic shape of the NCs regardless of the K^+^/Rb^+^ doping, as shown in Figure 2b–d. We investigated that K^+^ can bond with the bromide ions in a solution of NCs to form a protective inorganic ligand layer outside the NC surface. Therefore, the reduced amount of organic ligands could be a primary reason for the increased size of the NCs,^18^ where the average size of pristine and K-doped NCs is 10.72 and 12.36 nm, respectively. After 3% Rb^+^ doping, the average size of the NCs decreases to 9.58 nm. Because of the smaller size of the Rb^+^ ions, the *d*-plane spacing between crystallographic planes is reduced, and the size of the Rb^+^-doped FAPbBr_3_ NCs decreases.^13^

**Figure 3:**
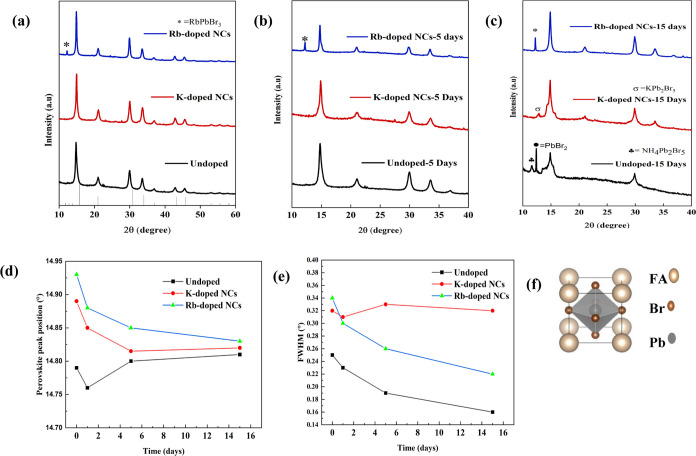
XRD pattern of (a) freshly prepared thin films on the glass substrate. (b, c) XRD patterns of all samples exposed to a humid environment for the stated times. The features are assigned as stated; we assign the feature marked * to be the nonperovskite phase of RbPbBr_3_. (d) Peak position and (e) fwhm for the perovskite NCs over time. (f) Basic cubic crystal structure of FAPbBr_3_ NCs at room temperature.

Accordingly, X-ray diffraction (XRD) measurements are performed to further explore the structure of PeNCs and to observe the formation of perovskite and nonperovskite crystal phases after aging under laboratory ambient conditions. We stored the pristine perovskite samples at a constant room temperature of 298 ± 1 K and a relative humidity of 25 ± 3% for 15 days. The main X-ray diffraction peaks for doped and undoped samples (Figure 3a,b) located at 14.79, 21.20, 29.98, 33.52, 36.85, 42.84, and 45.53° corresponding to the crystal planes (100), (110), (200), (210), (211), (220), and (300), respectively, confirm the cubic phase of FAPBr_3_ with the *Pm*3̅*m* space group. These results are consistent with the cubic structure JCD 87-0158 and with those reported elsewhere.^15,19^

As shown in Figure 3c for the undoped NCs, we observed an intense PbBr_2_ peak at 2θ = 12.3° after storing samples under ambient conditions for 15 days. The humidity exposure for undoped samples also leads to the formation of a new reflection with the peak at 2θ = 11.6° that we assign to the undesired white-phase NH_4_Pb_2_Br_5_.^20^ NH_4_^+^ could form by the decomposition of FA^+^ during the storage of samples. For the Rb-doped perovskite samples (Figure 3a–c), we found a different diffraction peak at 2θ = 12.25° that we describe as the nonperovskite phase of RbPbBr_3_.^21^ We observe that there is a systematic increase in the intensity of the diffraction peak at 2θ = 12.25° with a storage time of up to several days. The X-ray patterns correspond to K-doped NCs exposed for 15 days and contain a weak reflection at 2θ = 12.86° that may depict the nonperovskite phase of KPb_2_Br_5_ (Figure 3c). Figure 3d,e demonstrates the variation in the primary diffraction peak at 2θ ≈ 14.80° and the full width at half-maximum for the doped and undoped samples at different exposure times at ambient conditions. From this study, we can see that the peak position of the K- and Rb-doped NCs is shifted toward a higher angle relative to the undoped NCs, as smaller ions cause lattice contraction. This interpretation is consistent with the recent literature on K and Rb doping for stable and efficient perovskite devices.^18,22^ We note that we have not realized any significant change in the peak position after humidity exposure, proposing that these properties are not affected by ambient conditions. We noted that the fwhm for undoped and Rb-doped NCs drops significantly, which is in agreement with the previously mentioned report.^23^ By contrast, these parameters remain similar for K-doped NCs after exposure to ambient conditions. Figure 3f presents the typical cubic structure of the FAPbBr_3_ NCs.

**Figure 4:**
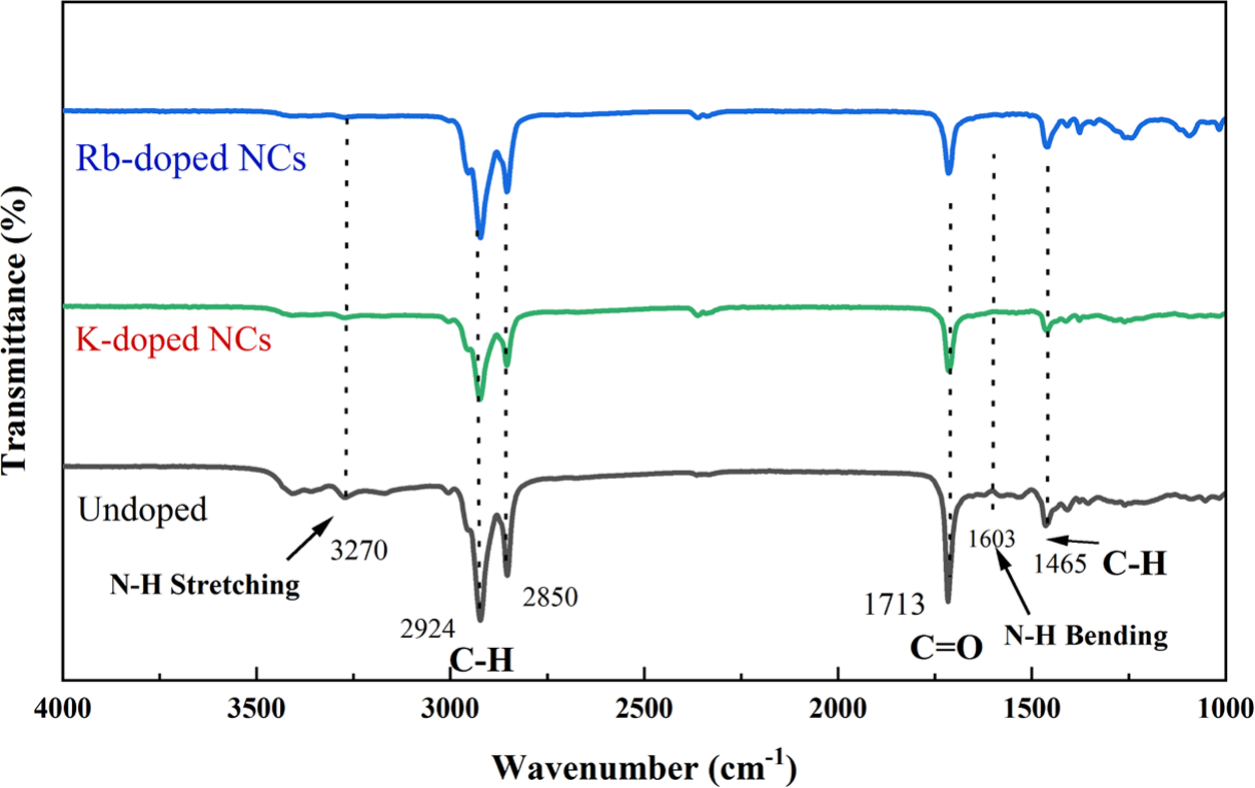
FTIR spectra of undoped, K-passivated, and Rb-passivated FAPbBr_3_ NCs.

We conducted Fourier transform infrared (FTIR) spectroscopy to further investigate how K^+^ and Rb^+^ influence the surface chemistry of perovskite NCs. By comparing the data with and without alkali metal ion doping (Figure 4), we notice that the peaks at 3270 and 1603 cm^–1^ ascribed to N–H stretching and N–H bending are significantly reduced with K^+^ and Rb^+^ doping, which specifies the reduced density of oleylamine to the outer boundary of FAPbBr_3_ PeNCs. These findings are consistent with that of Yang, who also found similar results, which show that the density of organic ligands is reduced, showing the reduced amount of surface ligands in perovskites.^18^ In addition, the strategy to introduce additional FABr to synthesize FABrPb_3_ NCs with fewer defects can also reduce the amount of insulating organic ligands.^15^ The strong peaks at 2700–3000 and 1713 cm^–1^ correspond to the C–H bending vibration and the C=O stretching of oleic acid, respectively. The reason for the reduction in ligand coverage could be attributed to K^+^/Rb^+^, which can make a bond with the halide ions in the colloidal solution of NCs to form an inorganic ligand-rich environment outside the NC surface.

**Figure 5:**
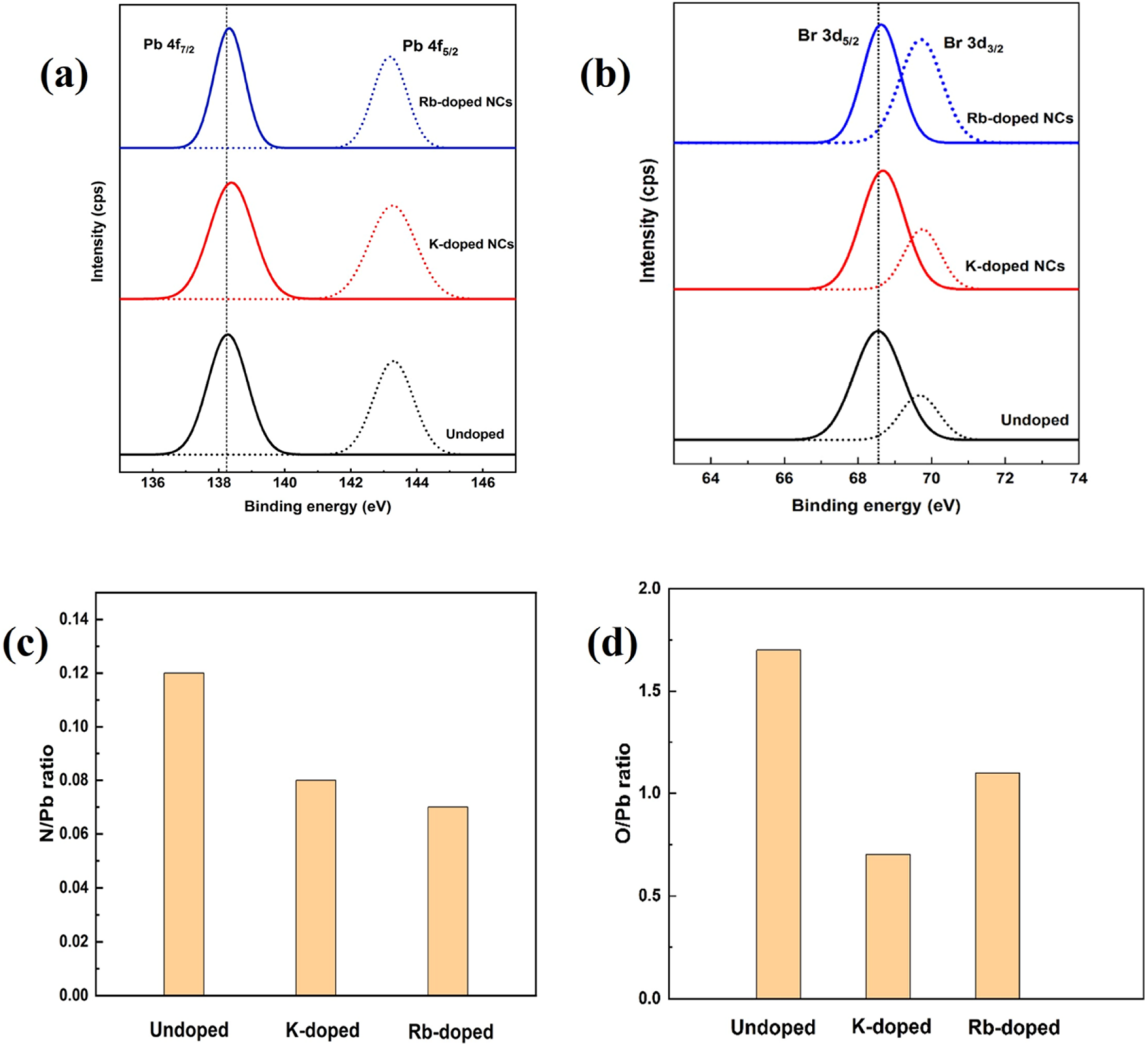
XPS spectra of (a) Pb 4f and (b) Br 3d peaks. (c) N/Pb and (d) (O/Pb) ratios of all samples of FAPbBr_3_ NCs.

Furthermore, X-ray photoelectron spectroscopy (XPS) spectra were recorded to recognize the chemical states of different elements with or without alkali metal ion-doped NCs. As shown in Figure 5a, the XPS characteristic peaks of Pb 4f_7/2_ and Pb 4f_5/2_, which were measured from undoped NCs, were recognized at 138.4 and 143.8 eV, respectively.

Interestingly, these peaks were found to show a shift toward a higher binding energy as the NCs were treated with K^+^ and Rb^+^, showing a different chemical environment of the Pb element. Similarly, the XPS spectral peaks of undoped samples for Br 3d_5/2_ and Br 3d_3/2_ (Figures 5b and S2) were noticed at 68.7 and 69.7 eV, respectively, and the peaks also showed a higher binding energy shift with K- and Rb-doped samples. Furthermore, we calculated the O/Pb and N/Pb ratios from the XPS spectra (Figure 5c,d), which showed that the O/Pb ratio considerably declined from 1.7 to 0.7 with the addition of alkali metal ions. As the O element is only present in oleic acid, the amount of oleic acid evidently decreases. The reduced value of the N/Pb ratio from 0.11 to 0.07 indicates the reduced concentration of oleylamine outside the perovskite NCs.

### 3.2 Dynamics of Photoexcited Charge Carriers

**Figure 6:**
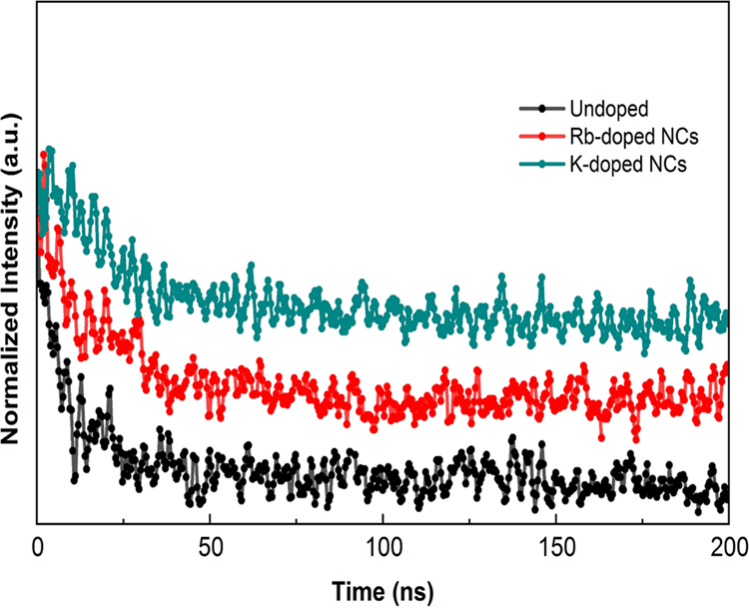
Time-resolved PL spectra for colloidal undoped, K-doped, and Rb-doped FAPbBr_3_ NCs.

From the data in Figure 6, this study shows the time-resolved PL to understand the dynamics of excitons and the generation and movement of the photoexcited charge carriers in the NCs treated with K^+^ and Rb^+^. The PL decay curves are well fitted with a biexponential function, which can be explained by the following equation:^24^
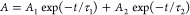
1where *A*_1_ and *A*_2_ are constants, *t* is time, τ_1_ is ascribed to the trap-assisted recombination, and τ_2_ represents the radiative recombination.

In previous studies on the kinetics of excitons, the PL decay times were measured as a function of faster decay, related to the trap-assisted recombination as an index of a shorter lifetime (τ_1_); slower decay, related to the radiative recombination as an indicator of a longer lifetime (τ_2_); and its fractional amplitude (*A*_i_).^19^ The average recombination lifetime (τ_ave_) was calculated from the fitted curve data according to the equation^24^
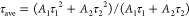
2

What is interesting about the data in Table 2 is that the τ_ave_ value increased from 45.6 ns of undoped FAPbBr_3_ NCs to 62.5 ns of K-doped samples. We find that the average lifetime decreased to 35.2 ns with Rb-doped FAPbBr_3_ NCs. These results found that an adequate amount of K^+^ can help to passivate the surface defects in the form of KBr, which inhibits halide phase segregation, contributing to a stronger radiative recombination. The enhancement of the radiative lifetime can also be explained by the inhibition of “Frenkel defects” when K^+^ stays at the interstitial site of the nanocrystal structure. In the case of Rb-passivated samples, the reduced average PL lifetime may be due to the presence of PL-inactive nonperovskite RbPbBr_3_, as reported in previous studies.^21,25^

**Table 2:** PL Lifetimes of Undoped, Potassium-Passivated, and Rubidium-Passivated FAPbBr_3_ NCs

alkali metal ion-treated samples	τ_1_ (ns)	*A*_1_ (%)	τ_2_ (ns)	*A*_2_ (%)	τ_ave_ (ns)
undoped	20.15	46.43	55.62	50.46	45.60
K-doped NCs	21.15	50.2	70.62	55.46	62.55
Rb-doped NCs	10.87	45.10	45.32	30.06	35.20

### 3.3 Passivation Mechanism of Alkali Metal Ions for Stable PeNCs

Theories in the literature regarding K^+^/Rb^+^ are extensive and focus particularly on the ion position inside the perovskite structure. Importantly, K^+^ moves into the interstitial position of the PeNCs to increase the structural stability, which impedes the exciton–phonon interaction and increases the thermal activation energy; hence, PeNCs continue to show a high emission intensity at high temperature.^10^ K^+^ has been reported as a highly effective passivator on the surface of lead halide PeNCs and binds with the halide ions to enhance the PL stability and suppress the halide ion segregation of the PeNCs.^2,26^ The definite location of both K^+^ and Rb^+^ is still an open question for researchers; however, recent published work proposes that neither K nor Rb are located within the perovskite lattice, yet there are also studies which reported that these alkali metal ions may take up the interstitial sites on the PeNC surface. Through density functional theory (DFT) calculations of kinetic properties of possible ion incorporation patterns, the researchers predicted that interstitial occupancy by alkali metal ions is indeed energetically more favorable than the A site cation substitution.^27^ In addition, DFT calculations also predict the increased ion migration barriers due to external alkali metal ion occupancy at interstitial sites. Also, the perovskite films can consolidate a higher amount of K^+^ than Rb^+^ before experiencing large-scale phase segregation.^23^ Rb^+^ addition into the perovskite films can stabilize the crystal phase, reduce surface traps, and increase the withstand voltage and efficiency of devices. The electrical characterization of Rb^+^-doped single-crystal PeNCs revealed the enhanced atomic interaction and orbital coupling between lead and halide ions, resulting in the increased carrier transport for X-ray detection performance.^13,14^

### 3.4 Effects of K^+^/Rb^+^ on the Colloidal Stability of PeNCs

**Figure 7:**
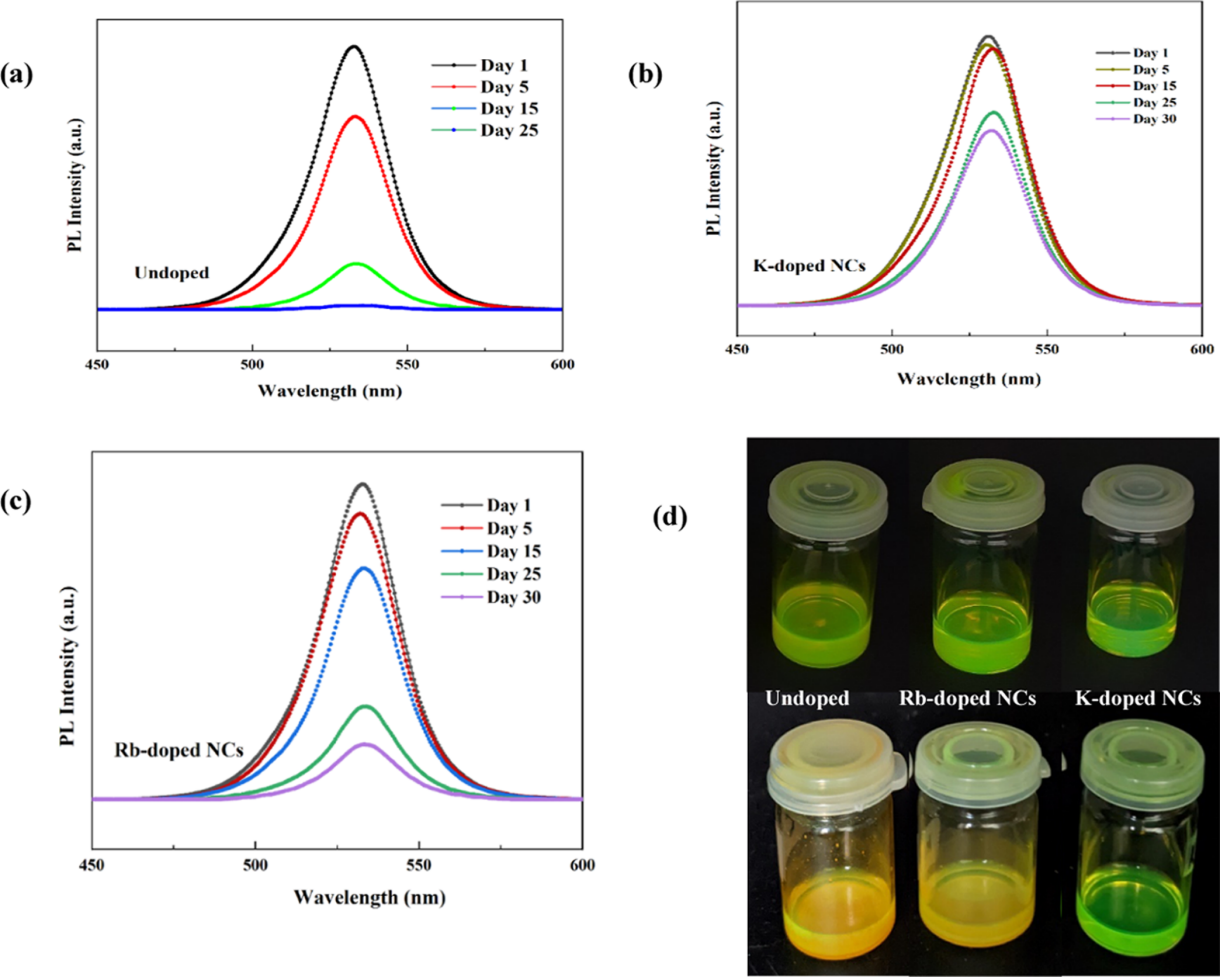
Room-temperature stability test of the FAPbBr_3_ perovskite NCs. (a–c) PL spectra of the dispersed undoped and doped FAPbBr_3_ NCs for 30 days. (d) Optical photographs of the corresponding perovskite NCs dispersed in hexane under 365 nm UV excitation.

One of the main limitations of the perovskite NCs is their inadequate colloidal stability because of the high surface energy of small-sized particles. Consequently, numerous passivating techniques have been widely explored to protect the PeNCs by using chemical ligands. Due to the liable chemistry of commonly used ligands including oleylamine and oleic acid, there is a weak interaction between the PeNC surfaces. These ligands are easily disjoined from the perovskite NC surface upon dilution or a long storage time; hence, structural damage or aggregation can occur. To test the effect of alkali metal ions on the stability of halide perovskites, we recorded the PL of the FAPbBr_3_ NC dispersion in hexane with and without K^+^/Rb^+^ doping. The results obtained from the FAPbBr_3_ NCs without doping show that the PL intensity reduced dramatically (Figure 7a), while K^+^-doped FAPbBr_3_ NCs show an insignificant change after 30 days of storage in ambient conditions (Figure 7b). Turning now to the experimental evidence from Rb^+^-doped FAPbBr_3_ NCs, an abrupt change in the PL intensity can be seen after 15 days of storage in air (Figure 7c). This finding could be the reason for the formation of the nonperovskite phase of RbPbBr_3_, as confirmed by XRD analysis. Figure 7d presents a comparison of undoped and K^+^/Rb^+^-doped FAPbBr_3_ NCs. For pristine and Rb^+^-doped FAPbBr3 NCs, the dispersion becomes cloudy after 25 days of storage, while K^+^-doped NCs retain their stability even after 30 days of exposure in ambient air. Due to a much stronger ionic bond, K-doped NCs show improved colloidal stability and reduced nonradiative recombination. For pristine and Rb-doped NCs, the NC dispersions become cloudy, which might be due to the reduced coverage of highly dynamic surface ligands and the formation of nonperovskite phases.

## 4 Conclusions

The alkali metal ion-doped FAPbBr_3_ perovskite NCs with green emission were synthesized by the ligand-assisted reprecipitation method under room temperature to reduce the surface defects. The doping strategy of K^+^/Rb^+^ was demonstrated to boost the PL emission intensity and the colloidal stability of the perovskite NCs. The chemical bonding of K^+^ with the bromide ions [Br^–^] in the solution of NCs to form an inorganic ligand surrounding outside the NC surface can help to passivate the nonradiative surface defects in the form of KBr, which inhibits the halide phase segregation, contributing to a stronger radiative recombination. Furthermore, K^+^ occupies the interstitial position of the NC structure, which increases the structural toughness and limits the dissociation of perovskite NCs, representing a remarkable stability for commercial use. The Rb^+^ doping tends to form the nonperovskite phase of RbPbBr_3_ with an average lifetime of 35.2 ns compared to 62.5 ns of K^+^ samples. To the best of our knowledge, the improved properties of FAPbBr_3_ perovskite NCs by alkali metal ion doping are rarely discussed research topics in halide perovskites. Further research work would be fruitful to fabricate low-cost and highly stable PeNC inks for optoelectronic devices.

## Supporting Information Available

Band gap calculation of PeNCs; experimental spectra of Pb 4f and Br 3d (PDF)
