# Supplementary material for: Fabrication of Potassium- and Rubidium-Doped Formamidinium Lead Bromide Nanocrystals for Surface Defect Passivation and Improved Photoluminescence Stability
Source: ACS Appl Electron Mater. 2024 Jan 4;6(1):550–8. doi: 10.1021/acsaelm.3c01542 (PMC10809411; doi:10.1021/acsaelm.3c01542)
Supplement: Supplementary file 1 — el3c01542_si_001.pdf [file el3c01542_si_001.pdf]

Supporting Information

Fabrication of Potassium- and Rubidium-doped Formamidinium Lead Bromide Nanocrystals for Surface Defects Passivation and Improved Photoluminescence Stability

Madeeha Tabassum[a], Qasim Zia[b], Huanqing Ye[c], William George Neal[d], Sameen Aslam[e], Jinshuai Zhang[f], and Lei Su[a*]

[a]*Key Labortary of Advanced Materials and Nanotechnology, School of Engineering and Materials Science, Queen Mary, University of London, London E14NS, UK; Email:*

[b]*Nano Vision Centre for structural and chemical analysis, School of Engineering and Materials Science, Queen Mary, University of London, London E14NS, UK*

[c]*The Photon Science Institute, Department of Electrical and Electronic Engineering, University of Manchester, Manchester M13 9PY, UK*

[d]*Centre for Condensed Matter and Materials Physics, School of Physical and Chemical Sciences, Queen Mary, University of London, London E14NS, UK*

[e]*Garments Technology Department, Punjab Tianjin University of Technology, Lahore 53720, Pakistan*

[f]*Key Labortary of Nanophononics and Semiconductor Optics, Materials Science and Engineering, Peking University, Yiheyuan Road, Beijing, 100871, China*

[*]Email: l.su@qmul.ac.uk

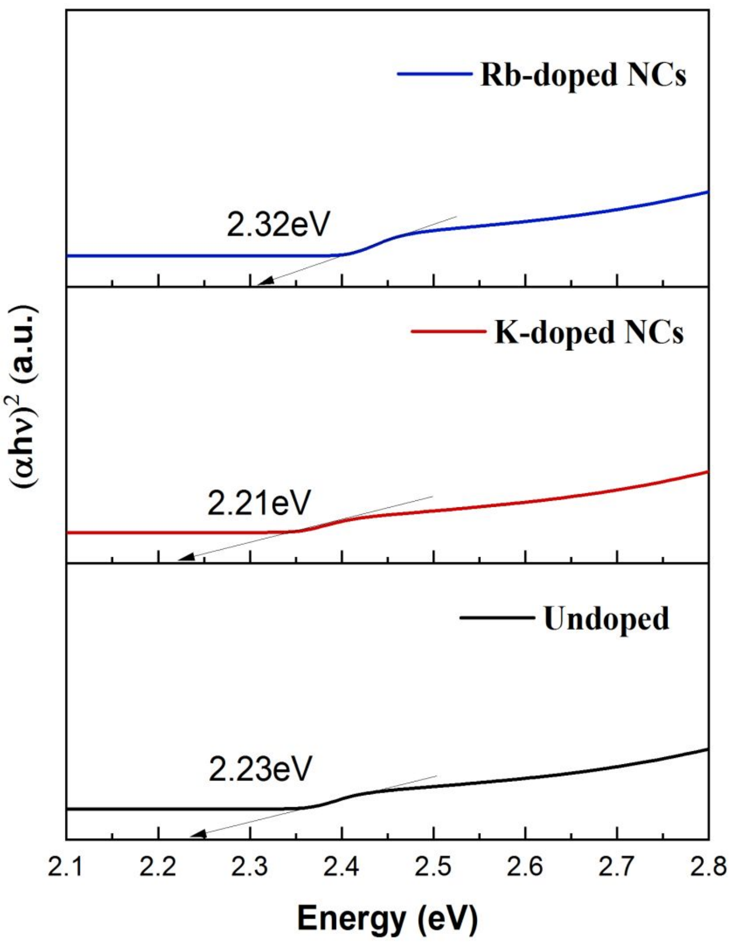

Figure S1. Band gap analysis of PeNCs using Tauc plots

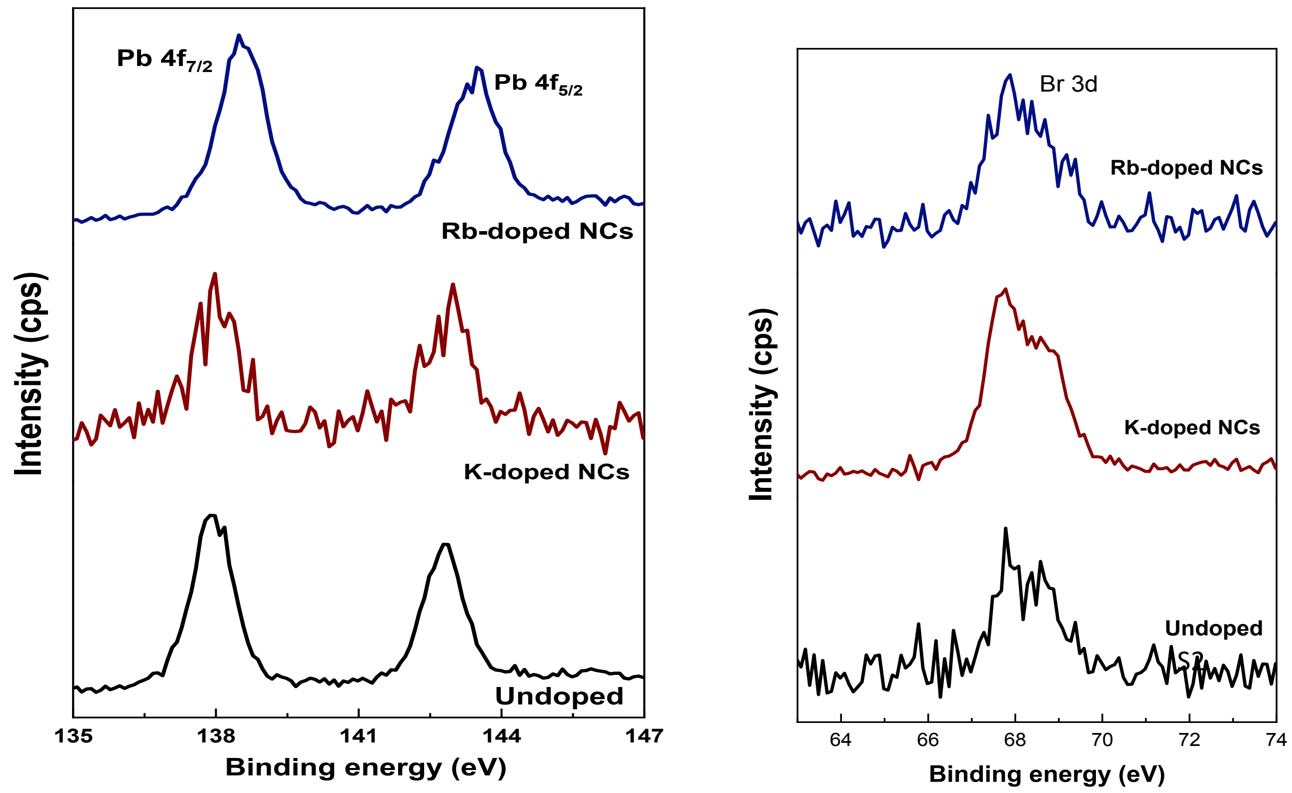

Figure S2. Experimental spectra of Pb 4f and Br 3d
